# Caregivers’ Experiences of Pathways to Care for Seriously Ill Children in Cape Town, South Africa: A Qualitative Investigation

**DOI:** 10.1371/journal.pone.0151606

**Published:** 2016-03-30

**Authors:** Caroline H. D. Jones, Alison Ward, Peter W. Hodkinson, Stephen J. Reid, Lee A. Wallis, Sian Harrison, Andrew C. Argent

**Affiliations:** 1 Nuffield Department of Primary Care Health Sciences, University of Oxford, Oxford, United Kingdom; 2 Division of Emergency Medicine, University of Cape Town, Cape Town, South Africa; 3 Primary Health Care Directorate, University of Cape Town, Cape Town, South Africa; 4 Department of Paediatrics, University of Cape Town, Cape Town, South Africa; University of Stellenbosch, SOUTH AFRICA

## Abstract

**Purpose:**

Understanding caregivers’ experiences of care can identify barriers to timely and good quality care, and support the improvement of services. We aimed to explore caregivers’ experiences and perceptions of pathways to care, from first access through various levels of health service, for seriously ill and injured children in Cape Town, South Africa, in order to identify areas for improvement.

**Methods:**

Semi-structured, qualitative interviews were conducted with primary caregivers of children who were admitted to paediatric intensive care or died in the health system prior to intensive care admission. Interviews explored caregivers’ experiences from when their child first became ill, through each level of health care to paediatric intensive care or death. A maximum variation sample of transcripts was purposively sampled from a larger cohort study based on demographic characteristics, child diagnosis, and outcome at 30 days; and analysed using the method of constant comparison.

**Results:**

Of the 282 caregivers who were interviewed in the larger cohort study, 45 interviews were included in this qualitative analysis. Some caregivers employed ‘tactics’ to gain quicker access to care, including bypassing lower levels of care, and negotiating or demanding to see a healthcare professional ahead of other patients. It was sometimes unclear how to access emergency care within facilities; and non-medical personnel informally judged illness severity and helped or hindered quicker access. Caregivers commonly misconceived ambulances to be slow to arrive, and were concerned when ambulance transfers were seemingly not prioritised by illness severity. Communication was often good, but some caregivers experienced language difficulties and/or criticism.

**Conclusions:**

Interventions to improve child health care could be based on: reorganising the reception of seriously ill children and making the emergency route within healthcare facilities clear; promoting caregivers’ use of ambulances and prioritising transfers according to illness severity; addressing language barriers, and emphasising the importance of effective communication to healthcare providers.

## Introduction

Reducing avoidable childhood morbidity and mortality is an international priority, particularly in sub-Saharan Africa[1] where there is an urgent need for research into the provision of care for the critically ill and injured[2]. Understanding pathways to care for seriously ill or injured children, from the decision to access health care through various levels of health services to the highest level of care, is a powerful yet under-utilised tool for tackling preventable morbidity and mortality. An established method for understanding patients’ experiences and perspectives is narrative, in-depth interviewing[3]. Caregivers’ narratives of their children’s experiences through the health system provide alternative and more personal perspectives than healthcare provider perceptions or clinical outcomes[4,5]. Qualitative research with patients/caregivers is therefore crucial for identifying aspects of health care which matter to them, improving evidenced-based, patient-centred care[6–9], and highlighting issues to be addressed to improve access to and quality of child health care[10–12].

South Africa (SA) has seen improvements in child mortality rates in recent years (under-5 mortality was 41 per 1000 live births and infant mortality 29 per 1000 live births in 2013)[13]. Yet avoidable health-system factors were estimated to contribute to a quarter to half of maternal, neonatal and child deaths[14]. Leading causes of child death include HIV/AIDS, pneumonia, sepsis, diarrhoea and injuries[14].

Health outcomes are dependent on good care at every level, and effective integration across the health system[15]. Indecision and delay may occur at all phases[16], thus a system-wide approach is necessary to highlight where improvements could be made, which could benefit critically ill children particularly in more resource limited settings with the greatest need[17].

We conducted an observational cohort study with an embedded qualitative component to describe the pathways to care of critically ill and injured children, from first presentation through to paediatric intensive care unit (PICU) admission or death, in Cape Town, SA. The observation cohort study aimed to quantitatively describe the details of the entire care pathway to PICU or death, and to use expert clinical review to evaluate the quality of care along the pathway and identify preventable failures in the care provided; 282 children/caregivers were included. Those quantitative methods and results are published (Reference PONE-D-15-39534R1). The current paper reports on an embedded qualitative study which explored caregivers’ experiences and perceptions of pathways to care. Our focus was on highlighting where improvements are needed and how they can be made. This adds a novel, important and previously under-researched perspective to the evidence base regarding care of critically ill children, thus providing evidence to support the improvement of child health care services in SA and worldwide.

## Methods

### Setting and participants

This study was centred at one of two public tertiary referral hospitals for the Western Cape Province’s 1.5 million children[18]. Children (defined as less than 13 years of age by the hospital admission system) are referred primarily from the Cape Town metropolitan area, whose paediatric population is approximately 930,000[18], by general practitioners, nurse-led clinics operating during office hours (clinics), doctor-led community health centres operating 24-hours (CHCs) and hospitals. Although prioritization of patients through triage is mandated in CHCs and hospitals (but often poorly applied to children and after hours[19]), there is no system for prioritization at clinics, where emergencies and routine care are seen largely on a first come, first served basis. Patients are transferred by a well-established emergency medical service (EMS) consisting primarily of road ambulances staffed by crews of varying qualifications.

For this study, all emergency PICU admissions were identified every second week between November 2011 and October 2012. Additionally, children who died before reaching PICU (in the Emergency Department at the tertiary referral hospital or those of other facilities in the referral area) were identified. Eligible caregivers were those whose children were less than 13 years, had a clear acute health care episode originating at home or in the community, spent fewer than 5 days in hospital before PICU admission, were not under palliative care services, and had caregivers who were able to communicate in the predominant SA languages. Caregiver here refers to an adult in the household who was primarily responsible for the care of the child.

### Interviews

A research nurse identified caregivers of eligible children admitted to PICU and informed them about the study, following which they were approached in person by one of three trained and experienced interviewers. Caregivers of children who had died prior to PICU admission were contacted by the interviewers directly. Written consent was obtained prior to all interviews. Participants were interviewed once, usually within 1–2 days of PICU admission, and 2–3 weeks after a death, in a private room at the hospital or at participants’ homes or workplaces.

An interview was conducted with the caregiver(s) of each child in the language of caregivers’ choice. Interviewers were two female and one male researchers with home languages Xhosa or Afrikaans and all fluent in English, with backgrounds in nursing or social sciences. They were all trained, the initial interviews were observed, and interview content was regularly reviewed by members of the research team to ensure high quality and consistency.

Following demographic questions, interviews were semi-structured using a standardised but flexible topic guide (see Table 1) which was pilot tested in 20 caregivers and amended and expanded accordingly. Caregivers were asked to describe the entire pathway to care for their child from when they first became sick until PICU admission or death. Interviews lasted up to one hour and were audio recorded. The interviewers took field notes, and later transcribed verbatim and subsequently translated each interview into English.

**Table 1:** Summary of interview topic guide.

**Background information**	
Demographics	Child age
	Caregiver age, marital status, education level, employment
	Household income
	Household composition
Social information	Address
	Living conditions
Health facility access	Nearest health facility
	Use of the EMS
**Main section about pathway to care (semi-structured)**	
Pathway to care	What happened from the onset of illness (signs, timing, help seeking)
	Health facilities visited (access, transport, timing, what happened, communication)
	EMS, ambulance transfers (timing, what happened, quality of service)
	Timeline of what happened for the entire illness
	Satisfaction (what was good about care received, what could be improved)
	Any other information

### Data analysis

Data analysis was led by a British researcher (CJ), with no prior knowledge of the findings from the wider quantitative study. The study was influenced by grounded theory, adopting several of the key components. Since a thorough qualitative analysis of all 282 interviews was not feasible, a maximum variation sample of transcripts was purposively selected using basic characteristics of the children and caregivers (child age, diagnosis, outcome at 30 days, and caregiver age, marital status, household income and primary language of family). Sampling continued until authors agreed saturation was reached.

Data were analysed using the method of constant comparison, facilitated by QSR NVivo 10. Two authors (CJ and SH) independently drafted coding schemes and identified themes according to interview content. The codes and themes identified by both were very similar, and were combined into a working coding scheme. Data were then coded by CJ and checked by SH. As analysis progressed, codes and themes were refined and added. The coding scheme and emerging themes were discussed and agreed by all authors. Themes were verified by the interviewers who agreed that salient issues arising from the interviews were represented, and that there were no misinterpretations of the data. Our analysis explored the diverse range of caregivers’ experiences and perceptions, and any discrepant cases. For reporting, themes are grouped chronologically into the main steps caregivers experienced once the decision to access care had been made–deciding where to seek help, access to care, and quality of care once accessed. We use a case study to illustrate the experiences of caregivers in more detail, in particular how the themes apply to a single participant and how different issues accumulate across the pathway to care.

The study was approved by the Faculty of Health Sciences Research Ethics Committee, University of Cape Town (HREC 211/2011); and the Oxford Tropical Research Ethics Committee, Oxford University (OXTREC 29–11), as well as the Western Cape Department of Health and the City of Cape Town. Written consent was obtained prior to all interviews, including for the research team to access children’s medical records. We have followed the consolidated criteria for reporting qualitative studies (COREQ)[20]. Data are not publically available due to the sensitive nature of the data.

## Results

### Participants

Caregivers of 282 children participated in the larger cohort study (response rate for the cohort study was 82%), and 45 of those interviews were included in this qualitative analysis (Table 2). Four of the included interviews were conducted with multiple caregivers per interview (the child’s mother plus father or grandparent), one was conducted with a relative who was not a parent, and the remainder with the child’s mother. Baseline characteristics and outcomes of the 45 included participants were similar to the overall cohort. Participants were poorer than the general Western Cape population, fewer lived in formal dwellings (36% versus 80%) and the proportion living in informal dwellings was greater (51% versus 18%)[18].

**Table 2:** Characteristics of the 45 children and caregivers included in the qualitative analysis.

Characteristics of children			n (%)
**Child gender**		Male	25 (56)
** **		Female	20 (44)
**Child age**		Neonate (Less than 1 month)	6 (13)
** **		Infant (1 month-1 year)	15 (33)
** **		1 year-5 years	16 (36)
** **		Over 5 years	8 (18)
**Diagnosis**		Trauma (road traffic accident/ burns/ other)	7 (16)
** **		Cardiac (congenital/ myocarditis/cardiomyopathy)	4 (9)
** **		Gastroenteritis	4 (9)
** **		Neurological (meningitis/ epilepsy)	4 (9)
** **		Respiratory disease (infective/ obstructive airway)	12 (27)
** **		Sepsis/ septic shock	7 (16)
** **		Other	7 (16)
**Outcome 30 days after interview**	Died	Prior to PICU admission	6 (13)
** **		In or after PICU admission	5 (11)
** **	Alive	Discharged home	24 (53)
		Remained inpatient	10 (22)
**Characteristics of caregivers**			
**Mother's age (years)**		Mean (standard deviation)	29 (7)
**Marital status**		Single	25 (56)
** **		Married/long term partner	20 (44)
**Monthly**		Less than $125	16 (36)
** household income (missing for 1 case)**		$125-$300	14 (32)
** **		Over $300	14 (32)
**Type of dwelling**		Informal	23 (51)
** **		Formal	16 (36)
** **		Other	6 (13)
**Language**		Xhosa	26 (58)
		Afrikaans	11 (24)
		Other South African (including English)	8 (18)

Table 3 summarizes the themes and subthemes within each of the major pathway steps. The results conclude with a case study illustrating how different issues accumulate across the pathway to care.

**Table 3:** Emergent themes and subthemes.

Step in the pathway to care	Themes	Subthemes
**Deciding where to seek help**	Communication	Impact of previous visits on decisions about subsequent visits
** **	Caregiver tactics	Bypassing or not bypassing lower levels of care
**Access to care**	EMS	Perceptions of ambulances, and actual speed of arrival
** **		Ability of ambulances to reach address
** **	Route through facilities	Pathway within facilities before being seen by a clinician
** **	Non-medical assessments	Non-medics assessing severity and helping/hindering access to clinicians
** **	Caregiver tactics	Demanding (quicker) help or not
**Quality of care**	EMS	Speed of EMS transfers
** **		Prioritisation of EMS transfers
** **	Caregiver tactics	Monitoring and pointing out deterioration
** **	Communication	Language barriers
** **		Perceived criticism
** **		Communication and support after a child death

### Deciding where to seek help

#### Communication

Upon deciding to seek medical help for their child, caregivers had a decision about which facility to visit. Decisions were influenced by experiences of previous healthcare visits, for example avoiding facilities perceived as unhelpful or critical. Some caregivers were intimidated by healthcare professionals. One mother was told by a nurse *“Get away this child is fresh…*. *Look at her she is fresh and has nothing*, *you just like bring children for nothing*.*”* She did not access help again until a neighbour urged her to, despite admitting: *“I was not hopeful at all*. *Even when I was at home I was constantly crying…*. *It was better not to be by her side ‘cause her condition was stressing me*.*” (child’s age group*, *diagnosis and outcome at 30 days*: *1–5 years*, *acute abdomen*, *discharged home)*.

Some participants felt that the facility or medication itself would not help:

“I became lazy to go to the clinic because I know they will give me this mixture again” (infant,gastro-enteritis, discharged home)

#### Caregiver tactics

Three–all non-Xhosa-speaking–caregivers who were dissatisfied with care received at lower level facilities bypassed lower levels of care by going directly to the tertiary referral hospital:

She hadn’t improved and that was the time I told my husband… “Now it’s too bad. We just have to drive to Red Cross one way.” Interviewer: You didn’t have a (referral) letter? Nothing. And I was received, when I came in here, when the nurse and the doctor saw my child–they had come in–they took my child… I think if I’d come here in the first place, they would’ve helped me long ago, honestly. (>5 years, TB meningitis, discharged home)

Contrastingly, two Xhosa-speaking caregivers expressed apprehension to bypass lower levels of care:

“We noticed that this eye thing is getting serious the eye was all swollen up as if it’s about to explode… so we told him that we will take him to the clinic tomorrow so that we can have a letter that is going to transfer him to [hospital] because they give you a hard time there if you go without a letter.” (: >5 years, meningitis, remained inpatient)

These limited data suggest that non-Xhosa speaking caregivers were more likely to actively bypass lower level facilities compared to Xhosa-speaking caregivers. (The predominantly black Xhosa-speaking population are generally lower socioeconomic status[18].)

### Access to care

#### EMS

Access to care was straightforward for trauma cases, where children were taken directly to a facility and seen by a healthcare professional quickly upon their arrival. Access for children with illness was more complex. Caregivers travelled to healthcare facilities largely by walking, taking a taxi, or hiring a car from a family member or friend. When children became ill during the evening/night, caregivers commonly decided to wait and seek care in the morning, for reasons including difficulty finding transport, despite the availability of 24-hour access facilities and the EMS. Whilst some caregivers did call the EMS, others did not consider using this service or know the number to call, and many had negative attitudes towards it, commonly believing ambulances are too slow to arrive:

“Ambulances themselves if you call it now, it will arrive tomorrow… when it finally comes the child might be dead already” (infant, shock/gastro-enteritis, discharged home)

An additional problem was that ambulances are unable (and/or unwilling for safety reasons) to find homes in informal settlements:

“it is difficult for it to come because we do not have street addresses. If you phone for it you must go and wait for it far on the main road.” (1–5yrs, pneumonia, discharged home)

When the EMS was called caregivers often described ambulances arriving quickly, indicating a mismatch between their perceptions and actual speed of arrival.

#### Route through facilities

Upon arriving at a healthcare facility, before seeing a clinician caregivers described having to (1) be let in by a security guard (2) see the clerk at reception and open a folder, and (3) wait in a waiting area, with access to the consulting area often controlled by security guards. Whilst most caregivers described knowing where to go within the facility, there was uncertainty amongst some:

“After waiting there for my child’s turn, I was told that I’m waiting at the wrong place so I had to leave. So I was told to go to ‘Trauma’. I wasted a lot of time waiting there whereas I was supposed to go to ‘Trauma’.”(neonate, viral pneumonia, discharged home)

Each required step through the facility could provide an obstacle to prompt access to healthcare:

“When I got there that guy who creates folders was asleep… he didn’t wake up… I begged [him] to wake up he finally woke up, he forced himself to work and he kept on sleeping but I kept on asking him to hurry up… I spent about 15 minutes trying to wake up that guy, then I had to wait in the chair” (neonate, septic shock, died in PICU)

“We sat, sat and sat and we were told to wait, we sat, sat and sat. After a while I got fed up… I went in and said “…If my child is very seriously ill what should I do, because my child is really very sick?” They said “Well then Mama, go and sit there.” The sister was saying that.… I sat, and sat and sat… I’m sure it was about four or past 4 to five… Yes we arrived at two.” (1–5years, gastric ulcer, discharged home)

“I was not happy about the clinic because when you get there even if your child is an emergency, you must sit down and wait until the sister comes. You cannot quickly rush and go straight ‘cause if you do that they shout at you. Also by the hospital it’s the same thing.” (1–5years, neuropathy, discharged home).

#### Non-medical assessments

Despite some children not being prioritised or seen quickly when caregivers felt they should have been, there were positive experiences in all levels of facility of seriously ill children being identified quickly. Clerks and security personnel both helped and hindered caregivers from gaining quicker access to clinicians, depending on their perceptions of illness severity and degree of caregiver concern:

“I spoke to a security [guard] that was sitting near the door. I asked her where I can get water as [my daughter] was asking for water. She directed me where to get water and I gave it to her. The security lady said to me “your child does not look well at all”… The security lady told me to go inside and not wait on the benches” (1–5 years, shock/gastro-enteritis, died prior to PICU admission)

Sometimes other patients/caregivers allowed children who seemed more seriously ill to go ahead in the queue:

“I saw that my child was getting worse but I did not know what to do. People on the queue had mercy on us they allowed us to go first on the line.” (infant, pneumonia, discharged home)

Contrastingly, one clerk tried to prevent a child from seeing a clinician altogether because at 11.00am it was too late. Hence a number of different non-medically trained people (security guards, clerks, patients/caregivers) assessed illness severity and decided whether children should be prioritised.

#### Caregiver tactics

The mother dismissed by the clerk at 11:00am expressed powerlessness:

“she is very sick however if they say they cannot attend to her, I have no choice.” (infant, septic shock, discharged home)

Other caregivers demanded (quicker) help by shouting, persuading security personnel to let them through, approaching clinicians directly, or bypassing facilities with long waiting times:

“My child was coughing badly by now. Time went on. Six o’clock went by, then seven, then eight, nine, ten, eleven then only at after 12 midnight was my child attended to… What happened is that I forced my way in… I realised that if I don’t do something nothing will help my child, hence I took my folder and went…” (neonate, viral pneumonia, discharged home)

“His body started to contract and there were a lot of people ahead of me. When he became stiff, I told the security, “I can’t take it anymore.” So, the security said, “You can go inside”.” (> 5 years, sepsis, died prior to PICU admission).

“And we took him to the [district/regional] Hospital and then they said I have to fill in a file and wait and wait. So, I just took my child and I came straight here [tertiary referral hospital].” (infant, acute asthma, discharged home)

Hence rather than being passive recipients of the healthcare system, at every level of facility there were cases of caregivers employing tactics to access care quicker. Others sat in turn and unless someone (usually non-medical) spotted that they needed urgent attention, were left to wait.

### Quality of care

#### EMS

An issue of key importance to caregivers was referral. Caregivers whose children had been quickly referred onward felt positively about this, even if the referring facility had done little else to help their child. When EMS was called for onward referral, caregivers described great variability in waiting times for transfers. Some transfers happened without delay:

“the ambulance didn’t take long wouldn’t lie. Even other patients were saying wow the ambulance will take long but it was quick to arrive” (1–5 years, myocarditis, died in PICU)

Dissatisfaction with delays appeared more common for inter-facility transfers than for call-outs to the community, with some caregivers concerned that their children seemed not to be prioritized according to illness severity. One such caregiver described how she and one nurse identified the child’s urgent need for transfer, but were unable to negotiate transfer on an earlier ambulance:

“I asked the sister, “When is the ambulance coming?” [She said] “No, they’re on their way. They’re on their way.” Every time an ambulance would arrive, but they wouldn’t take me… Okay, the one [nurse] checked on him a lot and they said… “Mummy don’t worry, but this child isn’t well. Can’t they send an ambulance quickly or if the next one comes, send you with it?” I said, “I don’t know sister…”” (>5 years, septic shock, died prior to PICU admission)

#### Caregiver tactics

Another important factor to caregivers was how well they perceived their children had been monitored. Many were monitored closely and caregivers felt positively about this aspect of their care; but some caregivers themselves pointed out deterioration to clinicians:

“I asked [the nurse] why the child was so swollen… I told her the child was swollen and that she was cold… She said she was going to call the doctors; 2 or 3 doctors arrived and started working on her… Yes, and after that, they said they were going to take her to ICU.” (1–5 years, septic shock, remained inpatient)

“I called the sister and said, “Sister, look, her hands are blue. Is the needle not a little too…” So she said, “Oh, I see, yes. I’m going to call the doctor now.” The doctor never came to check her hands.” (> 5 years, unknown likely sepsis, died prior to PICU admission). This again illustrates caregivers playing an active role throughout their child’s pathway to care, thereby achieving earlier specialist care.

#### Communication

The majority of caregivers described communicating with clinicians in a language they understood directly or via translators, with some reporting that the doctors *“explained everything nicely” (neonate*, *sepsis*, *discharged home)*. However it is important to note that a Xhosa-speaking caregiver *“couldn’t hear a thing” (neonate*, *septic shock*, *died in or after PICU)*, and another pointed out:

“I might say yes I understood what you were saying in English yet I didn’t… I will hear here and there and not everything. My wish is to understand everything I am told about my child” (infant, cardiomyopathy, discharged home)

Some caregivers experienced excellent communication; however some perceived criticism, particularly regarding the timing of presentation (not necessary or too late); or felt their concerns were not taken seriously:

“I feel that they do not trust what you as mother see in your child and what you tell them.” (1–5 years, neuropathy, discharge home)

Feeling intimidated prevented caregivers from communicating effectively with clinicians:

“She [nurse] said “Get away this child is fresh. Look at her she is fresh and has nothing, you just like bring children for nothing.” I never said anything, just kept quiet, I just can’t talk in such situations. I kept quiet and never answered back.” (1–5 years, acute abdomen, discharged home)

“I don’t know why I was not allowed inside… So I just told myself that I should give up and not cause any argument when my child is sick. So I must just accept what she is telling me” (neonate, septicaemia, died post-PICU)

There was sometimes insufficient communication after a child death: two caregivers were never told the cause of their child’s death, and some lacked support:

“nobody’s come to help me, to encourage me to talk about it and things like that” (>5 years, died prior to PICU admission, cause of death unknown)

### Illustrative case study

A case study (Box 1) illustrates how these themes and subthemes apply to a single participant, and how different issues accumulate across the pathway to care.

**Box 1: illustrative case study**

This account is given by the mother of a girl aged between 1–5 years. The interview was conducted in the mother’s language Xhosa, and she was aged between 30–35 years. The child’s diagnosis was myocarditis, and she died in PICU.

She started on Tuesday… [on Wednesday] I took her to the pharmacy and the pharmacist looked at her and gave us medication…

On Thursday she woke up still complaining about the stomach. I took her to [a clinic]. At the clinic they said maybe she’s got worms and they gave her worm medication… I left at 06:00 in the morning… I rushed to the clinic because I wanted to join the queue early because the clinic opens at 08:00… we took a taxi. We waited there until the clinic opened when the nurses arrived. We handed in our cards and got our folders. She was then seen by the sister. Because what happens you wait there watching sisters going up and down. I showed another sister that hey sister this child is not well and the sister said I must go to that side… indeed I went to the folder’s side and told them that hey it’s been a long time I arrived here. I was stressed now because this child is not well and she is complaining about it. I could see her stomach rising. I went inside and the sister helped me… then I was given medication. I think I left there around 15:00

I woke up on Friday and I didn’t go to work. I rushed to… a doctor [private general practitioner] because the [clinic I visited the day before is closed on] Friday… I wanted to be there by 06:00 because that doctor gets so many people… I arrived to that doctor around 07:00 because I had to change a taxi… I arrived to the doctor and the doctor was fast to see us. The doctor gave her an injection and pills

on [Saturday] morning she woke me up saying the stomach is painful now. She was sweating, water just running on her body… I just looked for a car and we went to [a community health centre]… By 06:00 I was already there because I left home at 05:00… When I went to the folder’s section the people who were there for overnight shift told me to wait for the 07:00 shift… that man said to me sit sister and wait for the 07:00 people. I told him that this child is not well what must I do because I want to go to that side but he said no wait. Indeed I waited for 07:00 and those people (a new shift) arrived and we handed in our cards and our names were called. There were three other people who came before me but they all said I must go first because they could see that this child is very sick. She was crying now. I realised that I cannot wait on this queue… the time must have been 08:00 now because I had to wait now on that queue as we were waiting to be seen by the doctor. I couldn’t wait in that queue. I spoke with the security and told him that “hey security this child is not well and I have been here for a long time now…” the security told me to go inside to the doctor. I went inside and headed straight to the doctor. The doctor put her in bed and started looking at her. I brought all the medications I had been getting on previous days. I explained to the doctor that I have been going to doctors. The doctor said there is nothing he can do I must wait for the ambulance

the ambulance didn’t take long I wouldn’t lie. Even other patients were saying wow the ambulance will take long but it was quick to arrive. We must have arrived [at the tertiary referral hospital] around 11:00 and the doctor saw her… The doctor gave her this pill and panado [paracetamol] and told me to go home… I took those things and left.

Hey by midday this child no. I told her father that hey I cannot sleep with this child because she is crying and she is crying for me because of the pain… we looked for a car and the car brought us [to the tertiary referral hospital] again… we hired a car… we came straight to that place where the ambulance had brought us… I didn’t wait. I just went straight to that place where the paramedic had taken me. I passed through the reception and went straight to that sister who had helped me earlier… I didn’t talk to anyone at reception I just went straight to that sister and told her that I am back again here is the child she is not well. I told her that I was seen by a doctor today. Indeed the sister went inside to ask that doctor… That doctor took her and placed her on the bed. He told us that he will admit her… different doctors were looking at her without knowing what is wrong with her… We went for a scan several time… doctor said she must be rushed to ICU… so that is how we got here [to PICU]

This mother experienced obstacles and delays, and employed various tactics in an attempt to access quicker care for her daughter. She was aware that her daughter was seriously unwell and persisted in attempting to access help despite multiple barriers.

## Discussion

### Main findings and implications

Caregivers’ experiences and insights offer a unique perspective on the health system and how critically ill children are handled. Many undocumented aspects, known only to the caregivers who accompany their children, are revealed in this study, highlighting issues which are relevant to improving the care of critically ill children in SA and other middle income countries.

### Caregiver tactics

Some caregivers subverted or negotiated the healthcare system to their advantage, playing a proactive role rather than being passive recipients. Schneider et al. refer to the inventive ways in which patients take advantage of gaps and manage the system as patient tactics[21]. Many patients bypass lower levels of care[14,22], particularly more affluent patients, suggesting there may be inequity in the system[23]. Our findings suggest cultural differences may exist in attitudes, with Xhosa-speaking (likely lower-socioeconomic status) caregivers being less confident to employ tactics. Importantly, tactical behaviours often led to quicker specialist help, indicating that children whose caregivers do not employ tactics may be at greater risk of preventable morbidity and mortality. Ethnic differences in child mortality exist in sub-Saharan Africa, likely due to social differences as well as economic inequality[24]. Further research is needed to examine differences in tactical behaviours and related health outcomes between groups.

### Non-medical assessments

Informally, non-medics–security guards, clerks, patients/caregivers–assessed illness severity and determined whether children should be prioritised. Although all 24-hour access facilities in the study are mandated to triage patients, a better system may be needed for early identification of seriously ill children[25–27]–such as training front staff in identifying such children[28,29].

#### Route through facilities

It was sometimes unclear to caregivers where to go and how to access emergency care, even once inside facilities. Similarly, long queues, lack of immediate attention for seriously ill children and delays after contacting the healthcare system have been reported elsewhere[10,11,30]. Reorganising the reception of seriously ill children and making the emergency routeing within healthcare facilities clear could reduce obstacles and provide a smoother pathway.

#### EMS

There was a mismatch between caregivers’ beliefs that ambulances are slow to arrive and speed of arrival in practice, especially when called to the community, highlighting that EMS responses exceed community expectations. Convincing caregivers of the efficacy of EMS response, as well as employing strategies for attending children when an ambulance cannot reach their home, may result in more caregivers using this service, potentially avoiding delays in child access to care. Caregivers’ negative perceptions of EMS may also reflect lack of trust in the EMS as part of the broader medical system–particularly given the perceived criticism experienced by some caregivers in relation to use of medical services–and highlight a need for effective communication and trust-building across all levels of health service.

There were a number of cases with apparently long waiting times for inter-facility EMS transfers, and transfers not being clearly prioritised according to illness severity. Long transfer time to intensive care in SA has previous been reported[31], and it has been suggested that referral mechanisms should be streamlined by improving communication and transport mechanisms[30]. The current system prioritizes calls from the public, with lower priority given to inter-facility transfers (where the patient is thought to be already receiving medical assistance) which may not always give children the priority they need, given the rapid and time-sensitive deterioration of critically ill children. Our results suggest that a better system of communicating acuity and prioritising paediatric EMS transfers is necessary.

#### Communication

Communication was satisfactory in many cases, but a key concern for some caregivers as shown previously in SA[32]. Perceived criticism, as well as long waiting times and dissatisfaction with care, led some caregivers to visit multiple facilities for the same illness episode, which prevents continuity of care and contributes to delay[33]. Although delay in seeking care has been cited as a major cause of child death in SA audits, Chopra and colleagues highlight that carers often had attended healthcare facilities before the terminal event, and that help-seeking was delayed due to suboptimal care and advice, and perceived rudeness[14]. Felt or enacted criticism also impacts caregivers’ help-seeking in well-resourced settings[34]. Our findings suggest that better provision is needed for overcoming language barriers, that caregivers’ understanding should be explicitly checked, and that clinicians need awareness of how caregivers may perceive criticism and how this influences the way they access further care and the information they provide. Also, a protocol is needed to provide information and support to caregivers after a child death.

#### Recommendations

These findings suggest several specific and some relatively easily effected interventions, which from caregivers’ points of view might improve and optimize the health system. They include reorganising the reception of seriously ill children, making clear the emergency routeing within each healthcare facility and providing a clear pathway for serious cases. Initiatives to promote appropriate use of EMS may reduce delays in critically ill children accessing care; and the system of communicating acuity and prioritising paediatric EMS transfers according to severity could be much improved. Awareness of the importance of effective communication, and the explicit checking of caregivers’ understanding, could be promoted amongst clinicians, and better provision for overcoming language barriers, and supporting bereaved caregivers, should be made available. Caregiver educational interventions could promote prompt and appropriate (re)consultation to the healthcare service.

#### Strengths and limitations

This is the first study to qualitatively explore the entire pathway to care for seriously ill children in SA. The data uncover where interventions are most needed, using a patient-centred approach relevant to many settings worldwide. The qualitative approach has enabled caregivers’ perspectives to be uncovered without being constrained by hypotheses or responses imposed by researchers. We included a varied sample and added cases until saturation was reached. Data analysis was led by an independent researcher without pre-conceptions of the SA health system, and codes and emerging themes were agreed amongst all authors and interviewers (including local clinicians).

The study was limited to children admitted to PICU or who died before reaching PICU: whether the same issues apply to the many more children seen in the health care system with varying severity of illness cannot be determined. In an effort to explore what improvements could be made, we focused on negative experiences within this sample. Participants may also have provided dramatic accounts or ‘atrocity stories’ which may not be factual or representative of general experience[9], and responses of those whose children had died may have been influenced by their grieving process (although we conducted interviews soon after PICU admission or death to prevent recall bias). Future research should explore the perceptions of clinicians as well as caregivers.

## Conclusion

The experiences of caregivers guiding their critically ill children into and through a health system presents a unique perspective, with many issues congruent to those perceived and measured by clinicians such as delays within health facilities and in the referral process, early identification of critically ill children, and quality of care. These data provide insights into the difficulty and uncertainty facing caregivers about where and how to access emergency care for their sick children, the strategies they use to overcome these barriers, and the importance of good communication.

This setting–the Western Cape of SA, where health services at all levels are overstretched and resources limited–represents one of the better scenarios found in this and other middle income countries. This report of the lived experiences of caregivers from the onset of illness to the highest level of care gives a clearer indication of the issues that need to be addressed in this context.
